# Activation of Pancreatic Stellate Cells Is Beneficial for Exocrine but Not Endocrine Cell Differentiation in the Developing Human Pancreas

**DOI:** 10.3389/fcell.2021.694276

**Published:** 2021-08-18

**Authors:** Jinming Li, Bijun Chen, George F. Fellows, Cynthia G. Goodyer, Rennian Wang

**Affiliations:** ^1^Children’s Health Research Institute, Western University, London, ON, Canada; ^2^Departments of Physiology and Pharmacology, Western University, London, ON, Canada; ^3^Department of Obstetrics and Gynecology, Western University, London, ON, Canada; ^4^Department of Pediatrics, McGill University, Montreal, QC, Canada

**Keywords:** human fetal pancreas, pancreatic stellate cells, human islet-epithelial cell clusters, transcription factors, cell differentiation

## Abstract

Pancreatic stellate cells (PaSCs) are non-endocrine, mesenchymal-like cells that reside within the peri-pancreatic tissue of the rodent and human pancreas. PaSCs regulate extracellular matrix (ECM) turnover in maintaining the integrity of pancreatic tissue architecture. Although there is evidence indicating that PaSCs are involved in islet cell survival and function, its role in islet cell differentiation during human pancreatic development remains unclear. The present study examines the expression pattern and functional role of PaSCs in islet cell differentiation of the developing human pancreas from late 1st to 2nd trimester of pregnancy. The presence of PaSCs in human pancreata (8–22 weeks of fetal age) was characterized by ultrastructural, immunohistological, quantitative RT-PCR and western blotting approaches. Using human fetal PaSCs derived from pancreata at 14–16 weeks, freshly isolated human fetal islet-epithelial cell clusters (hIECCs) were co-cultured with active or inactive PaSCs *in vitro*. Ultrastructural and immunofluorescence analysis demonstrated a population of PaSCs near ducts and newly formed islets that appeared to make complex cell-cell dendritic-like contacts. A small subset of PaSCs co-localized with pancreatic progenitor-associated transcription factors (PDX1, SOX9, and NKX6-1). PaSCs were highly proliferative, with significantly higher mRNA and protein levels of PaSC markers (desmin, αSMA) during the 1st trimester of pregnancy compared to the 2nd trimester. Isolated human fetal PaSCs were identified by expression of stellate cell markers and ECM. Suppression of PaSC activation, using all-trans retinoic acid (ATRA), resulted in reduced PaSC proliferation and ECM proteins. Co-culture of hIECCs, directly on PaSCs or indirectly using Millicell^®^ Inserts or using PaSC-conditioned medium, resulted in a reduction the number of insulin^+^ cells but a significant increase in the number of amylase^+^ cells. Suppression of PaSC activation or Notch activity during the co-culture resulted in an increase in beta-cell differentiation. This study determined that PaSCs, abundant during the 1st trimester of pancreatic development but decreased in the 2nd trimester, are located near ductal and islet structures. Direct and indirect co-cultures of hIECCs with PaSCs suggest that activation of PaSCs has opposing effects on beta-cell and exocrine cell differentiation during human fetal pancreas development, and that these effects may be dependent on Notch signaling.

## Introduction

The pancreatic epithelium gives rise to functionally distinct endocrine and exocrine cells through a well-orchestrated multi-step process. In the developing human pancreas, the 1st trimester is characterized by the proliferation of branched tubules into primitive acini, islets and ducts (Lyttle et al., 2008; Gittes, 2009). By the 2nd trimester of pregnancy, changes in pancreatic architecture include the appearance of parenchymous lobules containing small adult-like islets that include four endocrine cell types and a fine capillary network (Slack, 1995; Polak et al., 2000; Piper et al., 2004; Lyttle et al., 2008; Puri and Hebrok, 2010). Numerous studies have delineated the cascades of transcription factors within the epithelium critical for guiding human and rodent pancreas development (Slack, 1995; Piper et al., 2004; Lyttle et al., 2008; Gittes, 2009; Puri and Hebrok, 2010). One critical pathway is Notch signaling, which controls endocrine and exocrine fate commitment in the developing mouse pancreas during specific windows of development (Apelqvist et al., 1999; Shih et al., 2012; Seymour et al., 2020). However, there has been no study to date that has focused on the functional role of the surrounding pancreatic stellate cells (PaSCs) on the differentiation of pancreatic cells during human development, presenting a critical knowledge gap in understanding whether PaSCs are important for pancreatic organogenesis.

PaSCs are non-endocrine, mesenchymal-like cells that reside in the periacinar, periductal, perivascular, and peri/intra-islet regions of human and rodent pancreas (Apte et al., 2012). The embryological origin of PaSCs is still unknown. Since PaSCs are highly similar to hepatic stellate cells, it has been proposed that PaSCs may be derived from either mesenchymal, neural-ectodermal or hematopoietic stem cells (Mato et al., 2012). PaSCs exist in either a quiescent or active state. Quiescent PaSCs store retinoids in their cytoplasm, which can be used to distinguish PaSCs from normal fibroblasts, and express desmin and vimentin (Bachem et al., 1998). Quiescent PaSCs play a role in regulating acinar cell function, ductal and vascular pressures, and maintaining tissue architecture in the adult human and rodent pancreas (Bachem et al., 1998; Omary et al., 2007). In contrast, active PaSCs display myofibroblast-like morphology with a loss of retinoids from their cytosol and increased expression of αSMA, and produce large amounts of extracellular matrices, growth factors and cytokines in response to damaged pancreatic tissue for remodeling and repair (Bachem et al., 2006; Omary et al., 2007; Apte et al., 2012). Overproduction of these factors in active PaSCs can result in fibrotic tissue accumulation and lead to the development of pancreatic diseases, including diabetes (Hayden et al., 2007; Hong et al., 2007; Hayden et al., 2010). Although there are no reports of studies directly investigating the role of PaSCs in human fetal pancreas development, there is evidence that some secreted factors (e.g., TGFβ1 and CTGF) from activated PaSCs can contribute to the development of the rodent pancreas (Tulachan et al., 2007; Guney et al., 2011). These indirect factors and potential direct interactions between PaSCs and the developing pancreas compartment can therefore contribute to pancreatic cell commitment, although this has yet to be evaluated.

In the present study, the spatial and temporal localization patterns of PaSCs were examined in the human fetal pancreas from 1st to 2nd trimester of pregnancy (8–22 weeks of fetal age). Using isolated human fetal PaSCs, the possible effects of PaSCs on human fetal islet-epithelial cell cluster (hIECC) differentiation were investigated in *in vitro* co-culture systems. Here, we showed, using transmission electron microscopy and double immunofluorescence staining, that PaSCs can be identified based on their stellate-like morphology and noticeable foot-like processes, and that a high population of PaSCs in the developing human pancreas are located near ductal cells and newly formed islet cells during the 1st trimester of pregnancy. Co-culture of freshly isolated hIECCs and active PaSCs resulted in a significant decrease in the number of beta-cells and increased exocrine cell number. Suppressing the activation of PaSCs or Notch signaling resulted in enhanced beta-cell differentiation and a decreased number of exocrine cells in hIECCs, suggesting that Notch signaling may be one mechanism by which PaSCs regulate human fetal pancreatic cell differentiation.

## Materials and Methods

### Human Fetal Pancreatic Tissue Collection

Human fetal pancreases (fetal age 8–22 weeks) were collected according to protocols approved by the Health Sciences Research Ethics Board at Western University (London, ON, Canada), in accordance with the Canadian Council on Health Sciences Research Involving Human Subjects guidelines. Tissues were immediately processed for electron microscopy, immunohistology, RNA or protein extraction or culture, with a minimum of three pancreases per age group (Lyttle et al., 2008; Al-Masri et al., 2010; Li et al., 2014; Riopel et al., 2014).

### Isolation and Culture of PaSCs

Human fetal pancreases at 14–16 weeks of fetal age, a stage at which there is an enriched PaSC population, were carefully dissected from surrounding tissues and immediately digested using dissociation buffer containing collagenase V (1 mg/ml; Sigma-Aldrich Canada) followed by filtration through a 250 μm nylon mesh to yield fine cell-clusters. PaSCs were isolated from human fetal pancreases using an outgrowth method and cultured in Dulbecco’s modified Eagle’s medium (DMEM)/Ham’s F12 (1:1 v/v) containing 10% fetal bovine serum (FBS, Invitrogen, Canada), as described previously (Vonlaufen et al., 2010; Chen et al., 2015). The verification of PaSC purity was assessed after the 2nd passage of PaSC cultures by immunofluorescence staining and western blot analysis for stellate cell selective markers, growth factors and extracellular matrix proteins, as previously reported (Chen et al., 2015).

### Assessment of PaSC Viability and Activation in Response to ATRA

Cultured PaSCs at passage 3–5 were plated in 96- or 24-well culture plates with triplicate wells per group (Chen et al., 2015). PaSCs were exposed to all-trans retinoic acid (ATRA, Sigma, United States), an active metabolite of vitamin A that can suppress PaSCs activation, at concentrations of 0 (control), 50, 100, or 500 nM in culture medium for 24, 48, or 72 h (Li et al., 2014). PaSC viability was examined by incubating cells with 3-(4, 5-dimethylthiazolyl-2)-2, 5-diphenyltetrazolium bromide (MTT) solution for 2 h at 37°C. Cells were harvested, lysed using DMSO, and assayed for absorbance at 595 nm using a Multiskan^®^ Spectrum spectrophotometer (Thermo Labsystems, United States), as previously described (Wang et al., 2005; Krishnamurthy et al., 2008). Each experiment was conducted in triplicate wells with at least six repeat experiments per group. Data are expressed as cell viability (fold change vs. 0 nM or control). The activation of PaSCs was assessed by measuring the expression of αSMA, cell growth and extracellular matrix (ECM) protein synthesis. PaSC culture media were collected and stored at –80°C as conditioned medium to be used for human islet-epithelial cell cluster (hIECC) culture studies.

### hIECC/PaSC Co-cultures and Notch Inhibitory Studies

Pancreatic tissues (17–22 weeks fetal age) were pooled at the time of collection and digested to yield hIECCs, which contained epithelial progenitors (i.e., PDX1^+^/CK19^+^) and endocrine cells, as described previously (Li et al., 2006; Saleem et al., 2009; Al-Masri et al., 2010; Li et al., 2014). The isolated hIECCs were co-cultured with active PaSCs in CMRL1066 media containing 5% FBS (Invitrogen) for 48 h before being harvested for RNA extraction or immunohistological analysis. The control groups were cultured with no PaSCs present. *Direct co-cultures:* sub-confluent (80–90%) PaSCs in a 12-well plate were pretreated with or without 100 nM ATRA for 24 h before hIECCs were plated on top. *Indirect co-cultures:* hIECCs were plated on Millicell^®^ Inserts and co-cultured with sub-confluent PaSCs pretreated with or without ATRA (Supplementary Figure 1). *Conditioned media cultures:* hIECCs were treated with or without media from PaSCs (PaSC-conditioned media) diluted to 20% (v/v) with CMRL1066.

For Notch inhibitory studies, PaSCs were pretreated with either anti-human Delta-like ligand 4 antibody (DLL4, 5 μg/ml, BioLegend, United States) or DLL4 plus gamma secretase inhibitor DAPT (10 μM, Sigma), which prevents Notch receptor signal transduction (Pazos et al., 2017; Billiard et al., 2018). The control group was PaSCs treated with IgG1 antibody (BioLegend). This was followed by co-culture with hIECCs using Millicell^®^ Inserts for 48 h.

### Tissue Processing for Scanning and Transmission Electron Microscopy

*For scanning electron microscopy (SEM)*, human fetal pancreas samples were cut into 2 × 2 mm fragments and fixed with 2.5% glutaraldehyde, as described previously (Riopel et al., 2014). Samples were subjected to critical point drying and palladium-gold coating, then visualized using a 3400-N Variable Pressure Scanning Electron Microscope (Hitachi, Canada). Pancreases collected from 16 to 20 weeks of fetal age were processed for this analysis.

*For transmission electron microscopy (TEM)*, pancreata were fixed in 2.5% glutaraldehyde in 0.1 M phosphate buffer and post-fixed with 1% osmium tetroxide and embedded in araldite medium (Riopel et al., 2014). Semi-thin (500 nm) and ultra-thin (60 nm) sections were prepared. Silver-gold ultra-thin sections were stained with 2% uranyl acetate in ethanol and Reynolds’ lead citrate, then examined with a Philips 410 electron microscope (FEI, Hillsboro, United States) at 60 kgvolts (Riopel et al., 2014). Cells were classified as PaSCs by their stellate-like appearance with the presence of long foot-like processes. For counting the number of PaSCs in the developing human pancreas, 3–6 semi-thin sections per pancreas were quantified based on their star-like shape with foot-like processes structures, with at least three pancreas per age group examined. Data are expressed as the number of PaSCs per pancreatic section area (mm^2^).

### Immunofluorescence and Morphometric Analysis

Whole pancreases or cultured hIECCs wrapped in 2% (wt/vol.) agarose were fixed and embedded in paraffin (Lyttle et al., 2008; Al-Masri et al., 2010; Li et al., 2014; Riopel et al., 2014). Sections (4 μm) were cut throughout the entire length of the pancreas or cell blocks and stained with appropriate dilutions of primary antibodies (Supplementary Table 1). The co-localization of PaSC markers (desmin, αSMA) with transcription factors (i.e., PDX1), proliferation (Ki67) and pancreatic markers (i.e., insulin) in the developing human fetal pancreas were determined by double immunofluorescence staining. The percentage of proliferating PaSCs was examined by counting double positive cells through 12 random fields from head, middle and tail regions of each pancreatic section and expressed as the mean percentage of proliferating cells within the total desmin^+^ and αSMA^+^ cell populations. To assess the effect of PaSCs on pancreatic cell differentiation, the hIECCs cultured with PaSCs were stained for endocrine, exocrine, and transcription factor markers. The percentage of immunoreactive cells was obtained by counting 500–1,000 cells per section per experimental group, with a minimum of three repeat experiments per group (Al-Masri et al., 2010; Li et al., 2014). To verify the activity of PaSCs in response to ATRA suppression, PaSCs cultured on coverslips were *in situ* stained for ECM markers (Supplementary Table 1).

### Real-Time RT-PCR

RNA from pancreatic tissue and cultured hIECCs was extracted using either TRIZOL reagent or the RNAqueous-4PCR kit (Invitrogen) (Lyttle et al., 2008; Al-Masri et al., 2010; Li et al., 2014). Real-time PCR analyses were performed using the iQ SYBR Green Supermix kit (Bio-Rad, Canada) and the primers listed in Supplementary Table 2. Relative gene expression was calculated and normalized to the internal standard, 18S rRNA, with at least three repeats per age or experimental group (Lyttle et al., 2008; Al-Masri et al., 2010; Li et al., 2014).

### Protein Extraction and Western Blot Analysis

Proteins from human fetal pancreases and cultured PaSCs were extracted in Non-idet-P40 lysis buffer. Equal amounts of protein were separated in 12% SDS-PAGE gels, transferred to a nitrocellulose membrane (Bio-Rad), and incubated with appropriate dilutions of primary antibodies (Supplementary Table 1) followed by the application of horseradish-peroxidase-conjugated secondary antibodies (Cell Signaling, United States). Proteins were detected using ECL-Plus western blot detection reagents (Perkin Elmer, United States). Bands were imaged and densitometry was quantified using the Versadoc Imaging System (Bio-Rad) (Al-Masri et al., 2010; Li et al., 2014).

### Statistical Analysis

Data are expressed as mean ± SEM. Statistical significance was analyzed using GraphPad Prism (version 8 GraphPad Software, United States) with 95% of confidence interval. The difference between two groups were analyzed by paired Student’s *t-*test, difference among more than two groups were analyzed by one-way ANOVA followed by Tukey’s *post hoc* test. Differences were considered to be statistically significant when *p* < 0.05.

## Results

### Characterization of PaSCs in the Developing Human Fetal Pancreas

**FIGURE 1:**
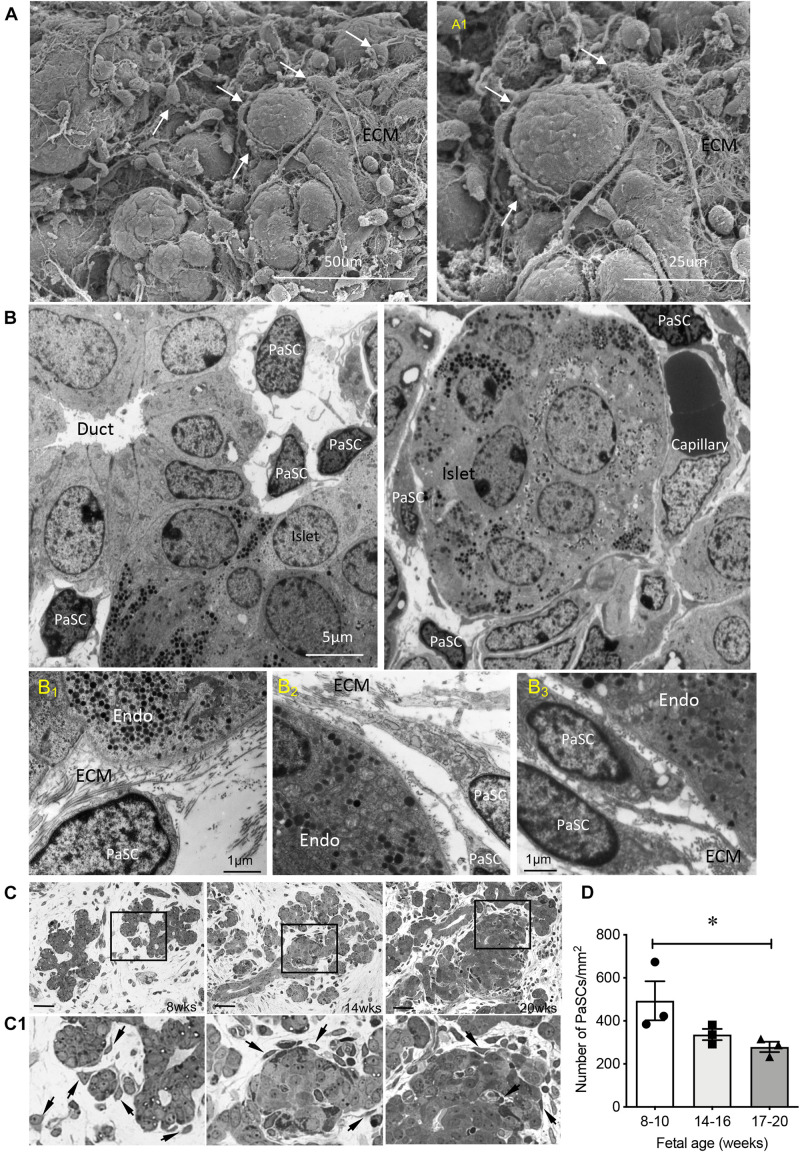
Ultrastructure and distribution of PaSCs in the developing human fetal pancreas. **(A)** Scanning electron micrographs of a human fetal pancreas at 20 weeks fetal age. PaSCs are identified by the presence of long foot process structures (arrows) surrounding cell clusters. Highlighted area is enlarged in **(A_1_)**. Scale bars = 50 μm **(A)** and 25 μm **(A_1_)**. **(B)** Transmission electron micrograph of PaSCs displayed long foot processes and are located near ducts with new endocrine cells budding off (14 weeks, left) and an islet surrounded by capillaries (20 weeks, right) in the human fetal pancreas. Representative images from 4 to 8 pancreata (14 and 20 weeks of fetal age) are shown. **(B_1–3_)** Highlighted transmission electron micrographs showed PaSCs and ECM fiber inter-connected to endocrine cells. Endo = endocrine cells, ECM = extracellular matrix. Scale bars = 5 μm **(B)** and 1 μm **(B_1–3_)**. **(C)** Representative images of PaSCs distribution on semi-thin (500 nm) sections from 8, 14, and 20 weeks pancreases, and **(D)** quantification of the number of PaSCs present in the developing pancreas. Scale bar: 100 μm. Magnified images are shown in **(C_1_)**, arrows indicate PaSCs. Closed circles, 8–10 weeks; closed squares, 14–16 weeks; closed triangles, 17–20 weeks. Data are expressed as mean ± SEM (*n* = *3* pancreata/age group). ^∗^*p* < 0.05, analyzed using one-way ANOVA with Tukey’s *post hoc* test.

**FIGURE 2:**
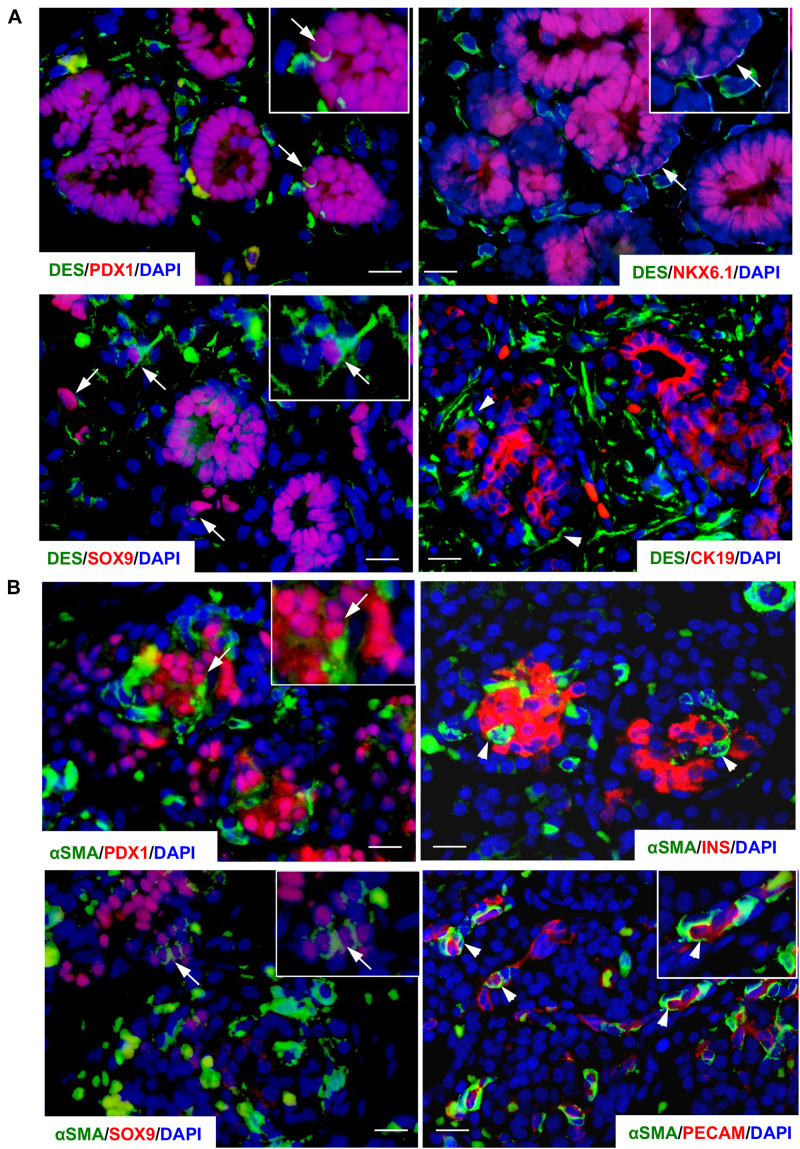
Co-localization of PaSC markers in the developing human fetal pancreas. Double immunofluorescence staining of PaSC markers **(A)** desmin (DES, green) with PDX1, SOX9, NKX6-1 or CK19 (red) in a 9-week human fetal pancreas; **(B)** αSMA (green) with PDX1, SOX9, insulin (INS) or PECAM (red) in a 20–21-week human fetal pancreas with the nuclear stain DAPI (blue). Scale bar: 25 μm. Arrows indicate co-stained cells and arrowheads identify single stained cells. Magnified images are shown in insets.

Distribution of PaSCs in the developing human pancreas was evaluated by scanning and transmission electron microscopy (Figure 1; Riopel et al., 2014). Based on their star (“stellate-like”) shape with foot-like processes, PaSCs were found to surround cell clusters (Figures 1A,A_**1**_), ductal cells and newly formed islets (Figure 1B), making complex cell-cell dendritic-like contacts. Higher magnification of the TEM showed PaSCs closely associated with extracellular matrix (ECM) around the endocrine cells (Figures 1B_**1–3**_). Using semi-thin sections (Figure 1C), the number of PaSCs was counted and showed that a high population was present during 1st trimester of pancreatic development followed by a significant reduction in later 2nd trimester (Figure 1D). Co-localization of PaSCs with transcription factors required for endocrine cell differentiation were assessed by double immunofluorescence staining. A few PDX1^+^, SOX9^+^ and NKX6-1^+^ cells were found to have positive labeling for desmin or αSMA, but the majority of the cells showed no co-staining (Figure 2), suggesting that PaSCs are not the primary progenitor cell source for endocrine cells in the developing human pancreas. Desmin^+^ or αSMA^+^ cells were located near CK19^+^, insulin^+^ and PECAM^+^ (CD31) cells with no co-localization (Figure 2), indicating that PaSCs could play a role to support cell differentiation during human fetal pancreatic development.

**FIGURE 3:**
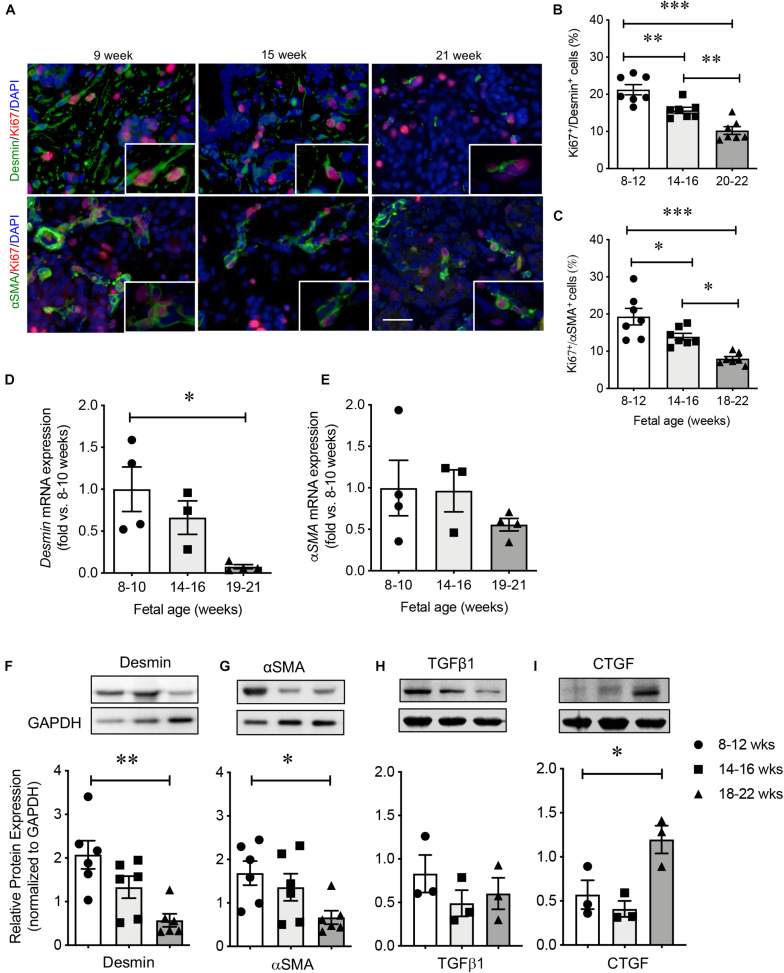
Characterization of PaSCs in the developing human fetal pancreas. **(A)** Representative double immunofluorescence staining images for desmin or αSMA (green) with Ki67 (red) and the nuclear stain DAPI (blue) in the human fetal pancreas. Scale bar: 25 μm. Magnified images are shown in insets. Quantification of proliferative PaSCs with Ki67^+^/desmin^+^ **(B)** or Ki67^+^/αSMA^+^ **(C)** in the developing human pancreas. qRT-PCR analysis of PaSC-related genes **(D)** desmin and **(E)** αSMA. Western blot analysis of proteins **(F)** desmin, **(G)** αSMA, **(H)** TGFβ1, and **(I)** CTGF. Closed circles, 8–12 weeks; closed squares, 14–16 weeks; closed triangles, 18–22 weeks. Data are expressed as mean ± SEM (*n* = 3–7 pancreata/age group). ^∗^*p* < 0.05, ^∗∗^*p* < 0.01, ^∗∗∗^*p* < 0.001; analyzed using one-way ANOVA with Tukey’s *post hoc* test.

High proliferative capacity of PaSCs was observed at 8–12 weeks of fetal age as indicated by high labeling of Ki67 in desmin^+^ and αSMA^+^ cells (Figure 3A). This was followed by subsequent declines at 14–16 and 18–22 weeks (Figures 3B,C). The mRNA levels of *DESMIN* and α*SMA* had high expression during early pancreatic development followed by a significant reduction at the 2nd trimester (Figures 3D,E), which was confirmed with microarray data from the developing human pancreas (Supplementary Table 3; Lyttle et al., 2008). Significantly decreased protein levels of desmin and αSMA were verified by western blot (Figures 3F,G). In contrast, there was no significant change in TGFβ1 and an increase in CTGF at 18–22 weeks (Figures 3H,I), both of which are reported PaSC products (Tulachan et al., 2007; Guney et al., 2011).

### ATRA Suppresses PaSC Viability and Production

**FIGURE 4:**
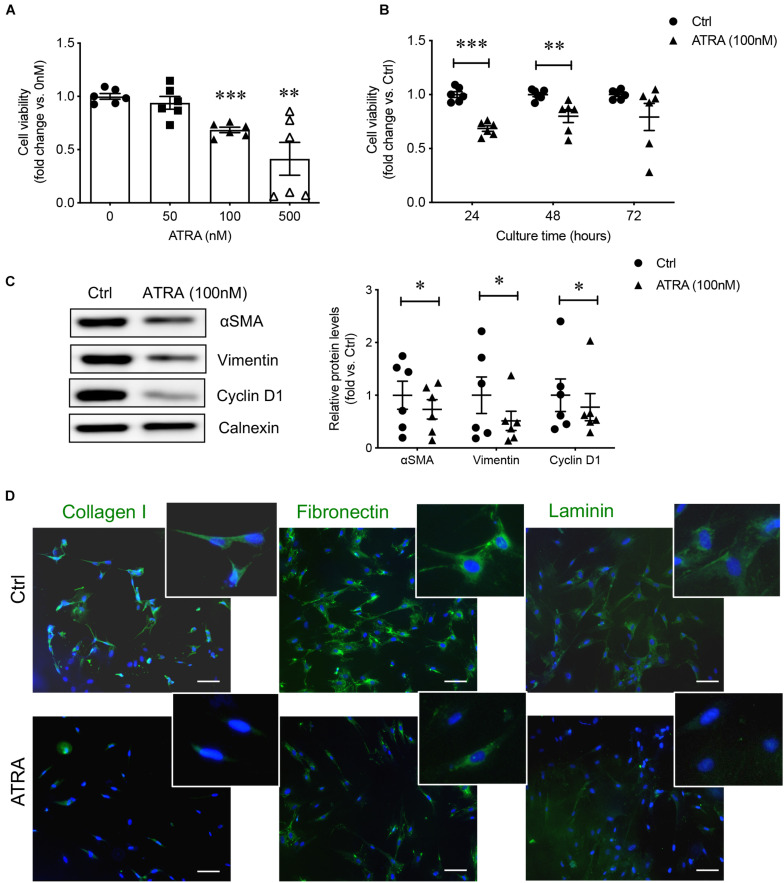
Activation of PaSCs was significantly reduced under 100 nM ATRA treatment. **(A)** Dose- and **(B)** time-dependent studies examining the effect of ATRA on PaSC viability by MTT assay. **(C)** Western blot analysis of activated PaSCs (αSMA, vimentin) and proliferation of PaSCs (cyclin D1) after 48 h treatment with 100 nM ATRA, normalized to the reference protein calnexin. Closed circles, control group; closed triangle, ATRA treated group. Data are normalized to control and expressed as fold changes (mean ± SEM, *n* = 4–6 experiments/treatment group). ^∗^*p* < 0.05, ^∗∗^*p* < 0.01; ^∗∗∗^*p* < 0.001 vs. control; analyzed using paired Student’s *t*-test. **(D)** Representative *in situ* immunofluorescence staining images of extracellular matrix proteins in cultured PaSCs of control and ATRA treated groups. Scale bar: 100 μm. Magnified images are shown in insets.

A pure preparation of human fetal PaSCs was used, where cells expressing PaSC markers were present while other contaminating pancreatic markers were absent (Chen et al., 2015). The phenotype of isolated human fetal PaSCs used in this study was compared to that of commercially purchased human PaSCs (HPaSteC catalog #3830, ScienCell Research Labs, United States) isolated from a 15-week human fetal pancreas and found to have similar PaSC phenotypes (Supplementary Figure 2). Treatment of active PaSCs with ATRA at 100 nM resulted in a significant reduction in PaSC growth efficiency as determined by MTT assay (Figure 4A), particularly after 24 and 48 h of treatment (Figure 4B). Loss of PaSC activity was also observed: there was a significantly reduction in αSMA, vimentin and cyclin D1 protein levels (Figure 4C) and decreased immunofluorescence staining for ECM proteins (collagen I, fibronectin, laminin) (Figure 4D). These data indicated that treatment of active PaSCs with 100 nM ATRA for 24–48 h is sufficient to significantly suppress PaSC activity and was used as a model of inactive PaSCs for subsequent studies.

### PaSCs Promote Exocrine Cell Differentiation During hIECC/PaSC Co-culture

**FIGURE 5:**
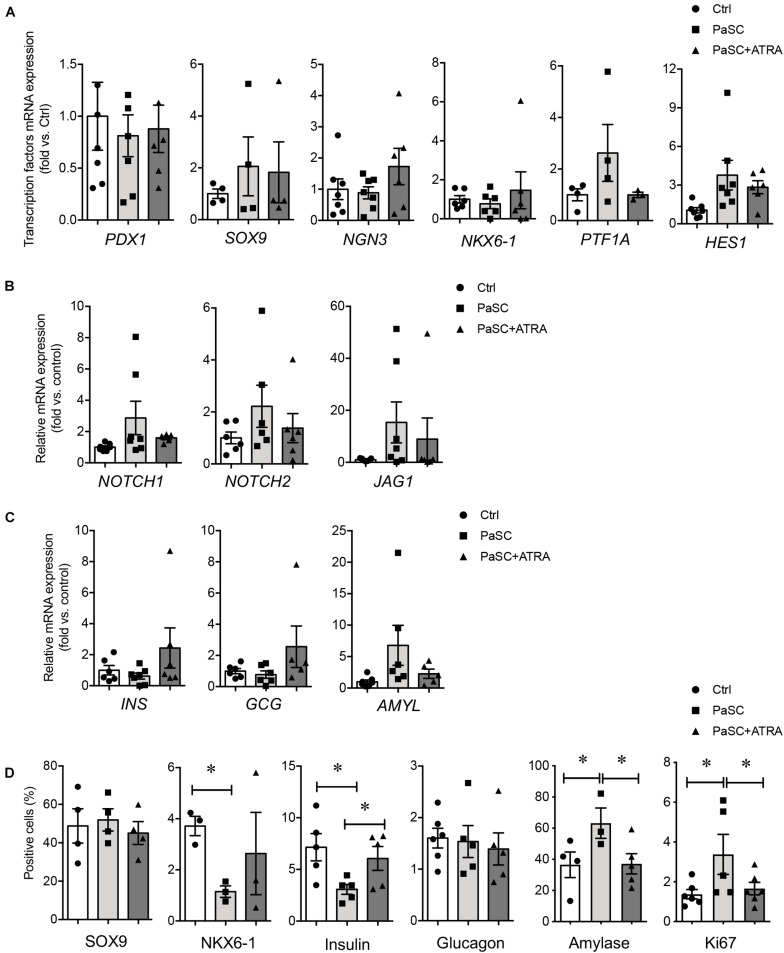
PaSCs promote exocrine cell differentiation in hIECCs during a direct co-culture. qRT-PCR analysis of **(A)** transcription factor, **(B)** Notch signals and **(C)** pancreatic genes expression, and **(D)** the percentage of SOX9^+^, NKX6-1^+^, insulin^+^, glucagon^+^, amylase^+^ and Ki67^+^ cells in hIECCs during a direct co-culture with PaSCs for 48 h. Closed circles, control (with no PaSC); closed squares, PaSC; closed triangle, PaSC + ATRA. Data are expressed as mean ± SEM (*n* = 3–7 experiments/treatment group). ^∗^*p* < 0.05; analyzed using one-way ANOVA with Tukey’s *post hoc* test.

Since the PaSC population was significantly reduced at 17–22 weeks (Figure 3), freshly isolated human islet-epithelial cell clusters (hIECCs) from 17 to 22-weeks fetal pancreata were used for co-culture studies (Saleem et al., 2009), which was mostly composed of pancreatic epithelial cells and contained only minimal desmin^+^ or αSMA^+^ cells (Supplementary Figure 3). To determine whether active PaSCs influence islet cell differentiation, hIECCs were co-cultured with PaSCs using three different methods. To assess the direct interplay between the cells, hIECCs were plated on top of cultured PaSCs. Although there were no statistically significant changes in gene expression levels, increased gene expression of the exocrine markers *PTF1A*, *HES1*, and *AMYL*, as well as *NOTCH* signaling, was observed in the hIECC/PaSC co-cultures (Figures 5A–C). The co-cultured cells also demonstrated a significant reduction in the percentage of NKX6-1^+^ and insulin^+^ cells, an increase in amylase^+^ cells, and no change in either the percentage of SOX9^+^ or glucagon^+^ cells (Figure 5D and Supplementary Figure 4), indicating that hIECCs are favored to differentiate into exocrine cells when directly cultured with active PaSCs. Suppression of PaSC activation by ATRA pretreatment followed by hIECC co-culture resulted in normalized beta-cell differentiation, as seen with the expression of endocrine cell markers and a reduced number of amylase^+^ cells in the culture (Figure 5D). High levels of cell proliferation (Ki67) were detected in the hIECC/PaSC co-cultures compared to hIECC/control and hIECC/PaSC + ATRA groups (Figure 5D).

**FIGURE 6:**
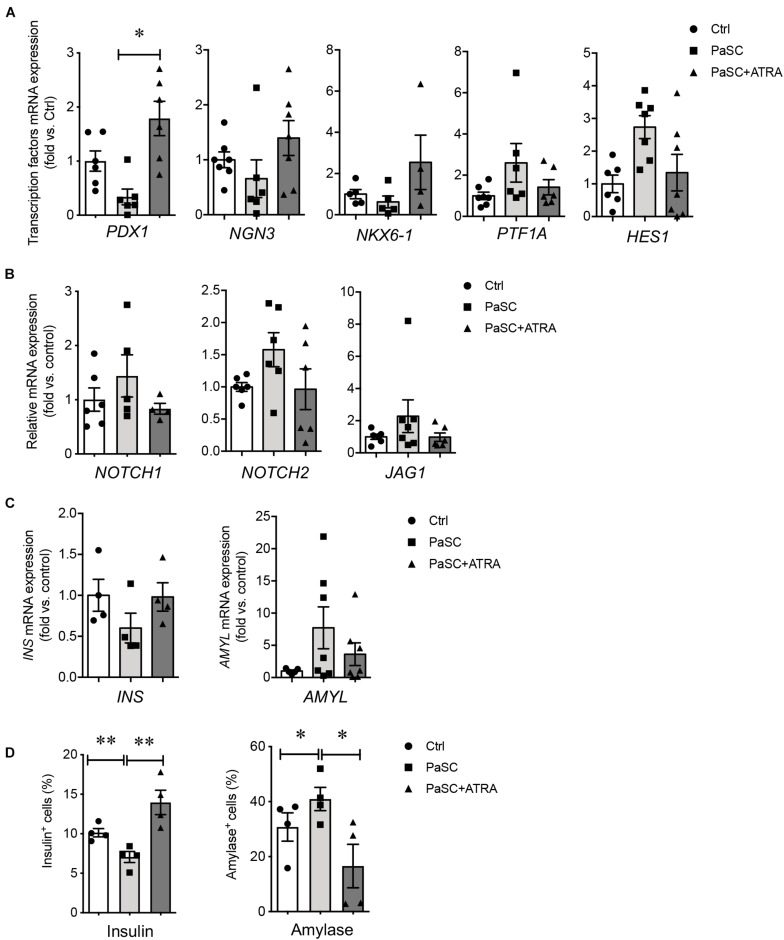
PaSCs enhance exocrine cell differentiation in hIECCs during an indirect co-culture using a Millicell^®^ Inserts culture system. qRT-PCR analysis of **(A)** transcription factor, **(B)** Notch signal and **(C)** pancreatic gene expressions, and **(D)** the percentage of insulin^+^ and amylase^+^ cells in hIECCs during an indirect co-culture with PaSCs for 48 h. Closed circles, control (with no PaSC); closed squares, PaSC; closed triangle, PaSC + ATRA. Data are expressed as mean ± SEM (*n* = 4–6 experiments/treatment group). ^∗^*p* < 0.05, ^∗∗^*p* < 0.01; analyzed using one-way ANOVA with Tukey’s *post hoc* test.

**FIGURE 7:**
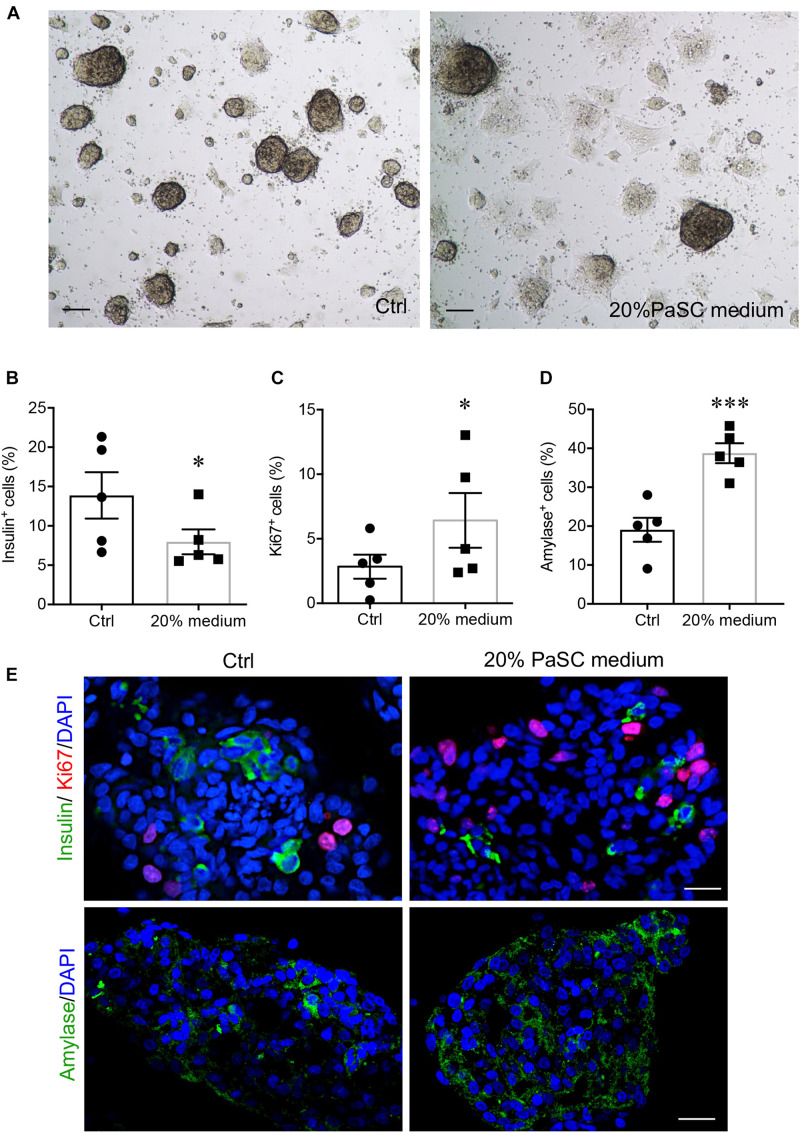
PaSC-conditioned medium enhances exocrine cell differentiation during hIECC culture. **(A)** Bright-field images of hIECCs cultured with or without 20% PaSC-conditioned medium for 48 h. Scale bar: 100 μm. The percentage of insulin^+^ **(B)**, Ki67^+^ **(C)** and amylase^+^ **(D)** cells in hIECCs. Closed circles, control; closed squares, 20% PaSC medium. Data are expressed as mean ± SEM (*n* = 5 experiments/treatment group). ^∗^*p* < 0.05, ^∗∗∗^*p* < 0.001; analyzed using paired Student’s *t*-test. **(E)** Representative immunofluorescence staining images for insulin or amylase (green) and Ki67 (red), and the nuclear stain DAPI (blue). Scale bar: 25 μm.

To characterize the indirect effects of active PaSCs on hIECC differentiation, a Millicell^®^ insert culture system was established, whereby hIECCs were loaded onto the insert and then co-cultured with PaSCs. Similar results from the direct co-culture experiment were observed with indirect insert hIECC co-culture, where no statistically significant changes were observed in gene expression levels (Figures 6A–C). However, there were significant decreases in insulin^+^ cells and increases in amylase^+^ cells compared to hIECC/control and hIECC/PaSC + ATRA groups (Figure 6D). These data from the indirect hIECC/PaSC co-cultures suggest that changes in islet cell differentiation may be due to PaSC-secreted factors. Further studies, using PaSC conditioned medium to treat hIECCs, were performed with similar outcomes to both co-culture studies (Figure 7). There was a loss of hIECC clusters with concomitant formation of monolayers during the 48 h co-culture period (Figure 7A), a significantly reduced insulin^+^ cell population, and a significant elevation in hIECC proliferation and number of amylase^+^ cells when compared to controls (Figures 7B–E). The PaSC secreted factor TGFβ1 has been shown to promote PaSC activation and cell proliferation (Supplementary Figure 5A; Kruse et al., 2000; Shek et al., 2002). To examine whether the effects of PaSC conditioned media on hIECCs may be due to TGFβ1, we added 10 ng/ml of TGFβ1 to hIECC cultures and confirmed that TGFβ1 decreased insulin ^+^ cell numbers similar to PaSC conditioned medium (Supplementary Figure 5B). These results suggest that PaSCs and their secreted soluble factors are beneficial for exocrine, but not endocrine, cell differentiation during the 2nd trimester of pancreas development.

### Blocking PaSC Notch Signaling Enhanced Beta-Cell Differentiation During hIECC/PaSC Co-culture

**FIGURE 8:**
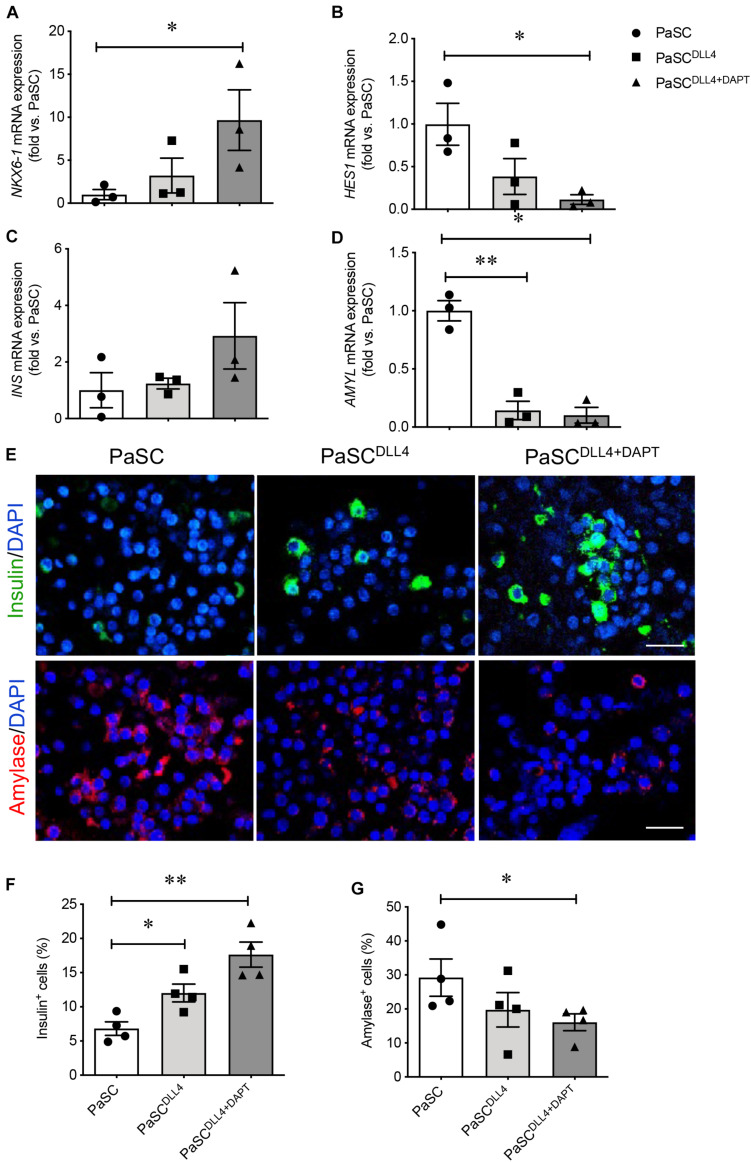
Blockade of PaSC Notch signaling enhances beta cell differentiation in hIECCs during an indirect co-culture. qRT-PCR analysis of transcription factors **(A)** *NKX6-1*, **(B)** *HES1*, and pancreatic genes **(C)** *INS* and **(D)** *AMYL*. **(E)** Representative immunofluorescence images for insulin (green) and amylase (red); nuclei were labeled with DAPI (blue). Scale bar: 25 μm. The quantification of **(F)** insulin^+^ and **(G)** amylase^+^ cells in hIECCs following an indirect Millicell insert co-culture with PaSCs pretreated with Notch inhibition (DLL4 antibody and DAPT). Closed circles, control (PaSC); closed squares, PaSC^DLL4^; closed triangle, PaSC^DLL4+DAPT^. Data are expressed as mean ± SEM (*n* = 3–4 experiments/treatment group). ^∗^*p* < 0.05, ^∗∗^*p* < 0.01; analyzed using one-way ANOVA with Tukey’s *post hoc* test.

Since Notch signaling in the developing mouse pancreas plays a key role for controlling the decision between endocrine and exocrine fates (Apelqvist et al., 1999; Shih et al., 2012; Seymour et al., 2020), it is possible that active PaSCs promote exocrine cell differentiation through Notch pathway in the developing human pancreas. Our previous microarray study for the developing human pancreas showed that Notch signaling genes in the human fetal pancreas are significantly reduced at the 2nd trimester (Supplementary Table 3). However, notable high levels of Notch signaling and marker expression was observed in hIECCs/PaSCs co-culture (Figures 5B, 6B). To test whether Notch signaling in PaSCs play a role in controlling exocrine cell differentiation during the hIECCs/PaSCs co-culture, a Notch signaling inhibitory study was performed using neutralized anti-DLL4 antibody or a combination of neutralized anti-DLL4 antibody and DAPT inhibitor. PaSCs were pretreated with these factors and then co-cultured indirectly with Millicell insert hIECCs. Although the hIECC/PaSC co-culture on neutralized anti-DLL4 antibody only induced mild changes in endocrine cell markers, the blockade of Notch signaling with neutralized anti-DLL4 antibody plus DAPT inhibitor demonstrated significantly enhanced NKX6-1 and increased *INS* gene expression (Figures 8A,C), with down-regulation of *HES1* and *AMYL* mRNA levels in the hIECCs (Figures 8B,D). Immunofluorescence staining verified a significant increase in the number of insulin^+^ cells along with reduced amylase^+^ cells (Figures 8E–G). These data indicate that, during human fetal pancreas development, PaSCs could have a negative effect on beta-cell differentiation and that this effect may be dependent on Notch signaling.

## Discussion

There is limited information about the presence of PaSCs in the fetal pancreas, although the role of PaSCs has been investigated extensively in the adult pancreas. These latter studies have shown that PaSCs found in periacinar, periductal and perivascular regions are responsible for regulating ECM turnover and tissue architecture in the adult human and rodent pancreas (Bachem et al., 1998; Omary et al., 2007). In the present study, we used multiple approaches to examine the spatial and temporal localization patterns of PaSCs in the developing human pancreas from 8 to 22 weeks. PaSC-related markers (desmin and αSMA) were highly expressed in a higher number of cells with enhanced proliferative capacity during early stages of pancreatic development and were reduced during the 2nd trimester of development, a critical window for human endocrine pancreas development (Lyttle et al., 2008). The location of PaSCs by scanning or transmission electron microscopy and double immunofluorescence staining clearly showed that labeled desmin^+^ and αSMA^+^ PaSCs could be readily distinguished from CK19^+^ ductal cells, CD31^+^ endothelial cells and insulin^+^ endocrine cells by their long foot processes distributed alongside ductal cells, capillaries and islet clusters (Chen et al., 2015). Their close contact with these areas of the pancreas may indicate that PaSCs have a role in supporting differentiation and/or function of these cells during human fetal pancreatic development, demonstrating that PaSCs may serve an important role in the differentiation of different cell compartments during human pancreatic development.

Data from hIECC/PaSC co-culture studies indicate that active PaSCs can diminish the number of insulin^+^ endocrine cells while favoring amylase^+^ acinar differentiation, and that secreted factors from PaSCs is one mechanism of action since conditioned medium reduced endocrine cell markers in hIECCs. Suppressing PaSC activation in the hIECC/PaSC co-cultures using ATRA resulted in increased endocrine cell differentiation together with a decrease in amylase^+^ cells, demonstrating that active PaSCs and their secreted soluble factors are important for the final outcome of exocrine and endocrine cell differentiation during human pancreas development. Co-cultured hIECCs with active PaSCs expressed relatively high levels of Notch signaling markers and blocking Notch signaling during the hIECC/PaSC co-cultures resulted in enhanced beta-cell differentiation, suggesting that Notch signaling may be one mechanism by which PaSCs regulate human fetal pancreatic cell differentiation. Collectively, these findings indicate that pharmacological modulation of PaSCs within human fetal islets could significantly impact pancreatic endocrine and exocrine cell differentiation.

It is notable that only a few transcription factors (e.g., PDX1^+^, SOX9^+^, NKX6-1^+^ and data not shown for other SOX^+^ transcription factors) were found to co-label with desmin or αSMA, suggesting that PaSCs may not be a primary progenitor cell source for either endocrine or exocrine cells in the developing human pancreas. The concept that PaSCs represent a progenitor or stem cell pool that can differentiate to other pancreatic lineages is controversial, as demonstrated in multiple publications (Lardon et al., 2002; Huang and Tang, 2003; Humphrey et al., 2003; Mato et al., 2009, 2012; Lucas et al., 2013). Studies of isolated nestin^+^ PaSCs from human fetal pancreatic islets demonstrated ABCG2 expression and were able to differentiate and assemble into islet-like clusters (Huang and Tang, 2003; Lucas et al., 2013). However, opposing reports have found that human fetal nestin^+^ cells failed to give rise to functional insulin-secreting beta-cells (Lardon et al., 2002; Humphrey et al., 2003). The divergent results may be explained by our previous study showing that nestin^+^ cells derived from neonatal rat islets represented a heterogeneous population, and that only a small subpopulation of nestin^+^/Pdx1^+^ cells was capable of forming insulin-producing cells (Wang et al., 2004).

Based on a modified PaSC isolation and purification protocol (Bachem et al., 1998; Vonlaufen et al., 2010), we successfully isolated human fetal PaSCs and verified the purity by positive staining for stellate cell selective markers (desmin, αSMA, vimentin) and negative staining for possible contamination from epithelial (CK19), endothelial (CD31) or endocrine (insulin) cells (Chen et al., 2015). The isolated PaSCs were verified against commercially available PaSCs to confirm the stellate cell-like phenotype (Supplementary Figure 2). Although we did not observe non-PaSCs in our isolation preparation, it is possible that a small population of non-stellate cells are present in PaSCs before hIECC/PaSC co-culture. To account for this issue, it is always recommended that co-culture experiments use the same preparation of both isolated hIECCs and PaSCs across different treatment conditions in case of the presence of contaminating cells within isolated PaSCs.

Unlike quiescent PaSCs, activated PSCs display a spindle-like shape *in vitro*, actively proliferate and migrate as well as show an increase in production of ECM proteins (Bachem et al., 1998; Chen et al., 2015). The recent use of single cell RNA-seq may provide additional distinguishing markers of PaSCs for future analyses, such as extracellular matrix proteins in activated PaSCs (Baron et al., 2016). ATRA, an inhibitor of PaSC activity, has been demonstrated by several groups to significantly reduce rodent PaSC proliferation, activation and ECM expression (Jaster et al., 2003; McCarroll et al., 2006; Froeling et al., 2011). A similar result was observed in the current study: human fetal PaSCs pretreated with ATRA displayed a significant reduction in cell proliferation with lower levels of ECM production, indicating that ATRA is sufficient to suppress human PaSCs activation *in vitro*. These data also correlate well with our previous findings showing that impaired PaSC function (by a knockout of β1 integrin on PaSCs in mice) resulted in a loss of ECM production, leading to decreased islet-acinar cell contact, acinar cell function and survival (Riopel et al., 2011; Riopel et al., 2013). Directly blocking β1 integrin on isolated human fetal PaSCs showed decreased PaSC migration, proliferation and associated growth factor levels (Chen et al., 2015). These findings show that activated PaSCs serve an important role in maintaining pancreatic tissue homeostasis.

PaSCs have been extensively studied in the adult pancreas as a major player in the development of pancreatic fibrosis, which can lead to several pancreatic diseases, including early islet fibrosis, the death of beta-cells and progression to diabetes (Yoon et al., 2006; Hong et al., 2007; Omary et al., 2007; Apte et al., 2012; Saito et al., 2012). Co-culture of PaSCs with RIN-5F cells resulted in a decrease in beta-cell survival and insulin secretion (Kikuta et al., 2013). However, the behavior of PaSCs in the developing human pancreas has been unclear. Using freshly isolated human fetal islet-epithelium cell clusters co-cultured with active PaSCs or treated with PaSC conditioned medium, we found enhanced exocrine cell differentiation and decreased insulin^+^ beta-cell differentiation, indicating that PaSCs and their secreted soluble factors are beneficial for exocrine but not endocrine cell differentiation during the 2nd trimester of human pancreas development. However, the hIECCs co-cultured with ATRA-suppressed PaSCs activation showed a significant enhancement of insulin^+^ beta-cell differentiation compared to hIECCs co-cultured with active PaSCs, which favored exocrine differentiation.

In the developing human pancreas, the ability of PaSCs to positively influence exocrine cell differentiation may be due to their secretion of large amounts of ECM and cytokines (Apte et al., 2012). Among the cytokines known to be secreted by PaSCs, TGFβ has been well documented to play a role in regulating pancreatic cell differentiation. Previous studies have demonstrated that blocking TGFβ signaling in the embryonic pancreas, using a transgenic mouse expressing a dominant-negative TGFβ-type-II-receptor, led to an increased number of endocrine cells arising from the embryonic ducts and increased proliferative capacity of the endocrine cells (Tulachan et al., 2007). TGFβ ligands have also been reported to display strong inhibitory effects on endocrine cell differentiation under *in vitro* test conditions (Guo et al., 2013). In our present study, we confirmed that adding TGFβ1 to hIECCs resulted in decreased numbers of insulin^+^ cells, which was a similar outcome when treating hIECCs with PaSC-conditioned medium. Taken together, these data support that PaSCs can shift the balance between endocrine and exocrine cell differentiation and that one mechanism may be through their secretion of TGFβ. Although this report has established that activated PaSCs can influence the lineage commitment of pancreatic cells within the developing pancreas, there are limitations on examining whether the co-culture of hIECCs with PaSCs impacted the function of developing endocrine and exocrine compartments since current co-cultures are too immature to further examine. Future studies should establish whether PaSC activation or inhibition can also impact beta cell maturation by examining endocrine maturation markers, such as MAFA and UCN3, or the acquisition of glucose-responsive insulin release in developing beta cells.

Both Notch and TGFβ signaling pathways play critical roles in the control of cell fate during pancreas development (Tulachan et al., 2007). Notch signaling occurs through cell receptor (Notch receptors) and cell ligand (Jagged, delta-like ligands) interactions within the developing pancreas, and regulated expression of these factors on early multipotent pancreatic progenitors are critical for determining early acinar, endocrine and ductal fate (Seymour et al., 2020). Activation of hepatic stellate cells by TGFβ1 could induce high expression of Notch pathway markers (Notch 1, Jagged1) and the transcription factor Hes1. However, this TGFβ1-induced stellate cell activation can be abolished by Notch blockade, indicating an interplay between Notch and TGFβ signaling (Aimaiti et al., 2019). Recent studies have confirmed that active Notch signaling were highly expressed in αSMA^+^ PaSCs, pancreatic intraepithelial neoplasia (PanIN) and tumor cells of mice with advanced pancreatic ductal adenocarcinoma (Plentz et al., 2009; Song and Zhang, 2018). The direct co-culture of PaSCs and human pancreatic cancer cells also led to dramatically enhanced cancer cell proliferation and activated the Notch signaling pathway in both cell types (Fujita et al., 2009). Blockade of Notch signaling inhibited PaSC activation, proliferation and migration, and reduced the PaSC-induced pro-tumorigenic effect and tumor progression (Plentz et al., 2009; Song and Zhang, 2018). The current study observed elevated Notch signaling and increased *HES1* gene expression during both direct and indirect co-culture of hIECCs with PaSCs, which resulted in promoting exocrine cell differentiation. Using a neutralized anti-DLL4 antibody plus DAPT inhibitor to block Notch signaling in PaSC cells prior to co-culture with hIECCs, we found enhanced *NKX6-1* and *INS* gene expression with down-regulation of *HES1* and *AMYL* mRNA levels in the hIECCs. In addition, there was an increase in the number of insulin^+^ cells paralleled by a reduction in amylase^+^ cells. These data support previous findings that Notch signaling can activate PaSCs by demonstrating an increase in exocrine cell commitment at the cost of endocrine cells, and that Notch blockade and inhibition in PaSCs can act as an indirect method of promoting endocrine cell fate in the developing pancreas.

## Conclusion

The present study is the first to provide insight into the spatial and temporal expression of PaSCs in the human fetal pancreas and sheds light on how PaSCs may be involved in endocrine/exocrine cell fate determination through TGFβ- and Notch-related mechanisms. These findings expand on how non-endocrine pancreatic cells present in the developing pancreas contribute to human islet commitment, and also suggest that manipulation of PaSC activity may prove to be a useful tool during the development of more effective cell-based therapies for the treatment of diabetes.

## Data Availability Statement

The original contributions presented in the study are included in the article/Supplementary Material, further inquiries can be directed to the corresponding author/s.

## Ethics Statement

The studies involving human participants were reviewed and approved by the Health Sciences Research Ethics Board at Western University (London, ON, Canada). The patients/participants provided their written informed consent to participate in this study.

## Author Contributions

JL and BC contributed to the acquisition of data, interpretation of results, preparation of the manuscript, revisions, and final approval for the revision to be published. GF and CG contributed to collect all human fetal pancreas used in this study, critical comments for manuscript and revision preparation, and final approval for the revision to be published. RW contributed to the experimental design, interpretation of data, the manuscript preparation, revisions, final approval for the revision to be published, and is the guarantor of this work. All authors contributed to the article and approved the submitted version.

## Conflict of Interest

The authors declare that the research was conducted in the absence of any commercial or financial relationships that could be construed as a potential conflict of interest.

## Publisher’s Note

All claims expressed in this article are solely those of the authors and do not necessarily represent those of their affiliated organizations, or those of the publisher, the editors and the reviewers. Any product that may be evaluated in this article, or claim that may be made by its manufacturer, is not guaranteed or endorsed by the publisher.

## Supplementary Material

Supplementary Figure 1 Co-culture models. Direct co-cultures **(A)** and Indirect co-cultures **(B)** models. Sub-confluent (80–90%) pancreatic stellate cells (PaSCs) in a 12-well plate were pretreated with or without 100 nM ATRA, then freshly isolated human fetal islet-epithelial cell clusters (hIECCs) were plated on top or in Millicell^®^ Inserts and co-cultured in CMRL1066 media containing 5% FBS for 48 h. **(C)** 20% Conditioned PaSCs media culture. **(D)** Indirect co-cultures with Notch inhibition.

Supplementary Figure 2 **(A)** Phase-contrast images of PaSCs isolated from human fetal pancreas in our lab and purchased HPaSteC (catalog #3830) from ScienCell Research Labs, United States. **(B)** Western blot gels present PaSC markers and growth factors of desmin, αSMA, vimentin, GFAP, TGFβ1 and CTGF from both cultured PaSCs and HPaSteC cells.

Supplementary Figure 3 **(A)** Freshly isolated human (18 weeks of fetal age) fetal islet-epithelial cell cluster (hIECC) containing a few PaSCs stained with desmin or αSMA (green, arrows); nuclei labeled by DAPI (blue). Scale bar: 25 μm. **(B)** The percentage of desmin^+^ and αSMA^+^ cells present in the hIECC was quantified, data are expressed as mean ± SEM (*n* = *5* experiments/treatment group).

Supplementary Figure 4 Representative immunofluorescence staining images for NKX6-1, insulin (red) or amylase (green) in hIECCs during a direct co-culture with PaSCs for 48 h, and the nuclear stain DAPI (blue). Scale bar: 25 μm. Magnified images are shown in insets.

Supplementary Figure 5 **(A)** PaSC viability by MTT showed a significant increase with 1–10 ng/ml TGFβ1 treatment. ^∗^*p* < 0.05, ^∗∗^*p* < 0.01 vs. 0 ng/ml. **(B)** The percentage of insulin^+^ cells in hIECCs cultured with 20% PaSC conditioned medium (closed squares) or 10 ng/ml TGFβ1 (open triangles) compared to controls (closed circles). Data are expressed as mean ± SEM (*n* = 3–5 experiments/treatment group).

Supplementary Table 1 Antibodies for immunohistochemistry, immunofluorescence and western blot analyses.

Supplementary Table 2 Sequences of primers used in Real-time RT-PCR assays.

Supplementary Table 3 List of genes for pancreatic stellate cell markers and associated notch signaling expressed during human fetal pancreatic development.
