# Supplementary material for: Activation of Pancreatic Stellate Cells Is Beneficial for Exocrine but Not Endocrine Cell Differentiation in the Developing Human Pancreas
Source: Front Cell Dev Biol. 2021 Aug 18;9:694276. doi: 10.3389/fcell.2021.694276 (PMC8418189; doi:10.3389/fcell.2021.694276)
Supplement: Supplementary Figure 1 — Co-culture models. Direct co-cultures (A) and Indirect co-cultures (B) models. Sub-confluent (80–90%) pancreatic stellate cells (PaSCs) in a 12-well plate were pretreated with or without 100 nM ATRA, then freshly isolated human fetal islet-epithelial cell clusters (hIECCs) were plated on top or in Millicell® Inserts and co-cultured in CMRL1066 media containing 5% FBS for 48 h. (C) 20% Conditioned PaSCs media culture. (D) Indirect co-cultures with Notch inhibition. [file Data_Sheet_1.PDF]

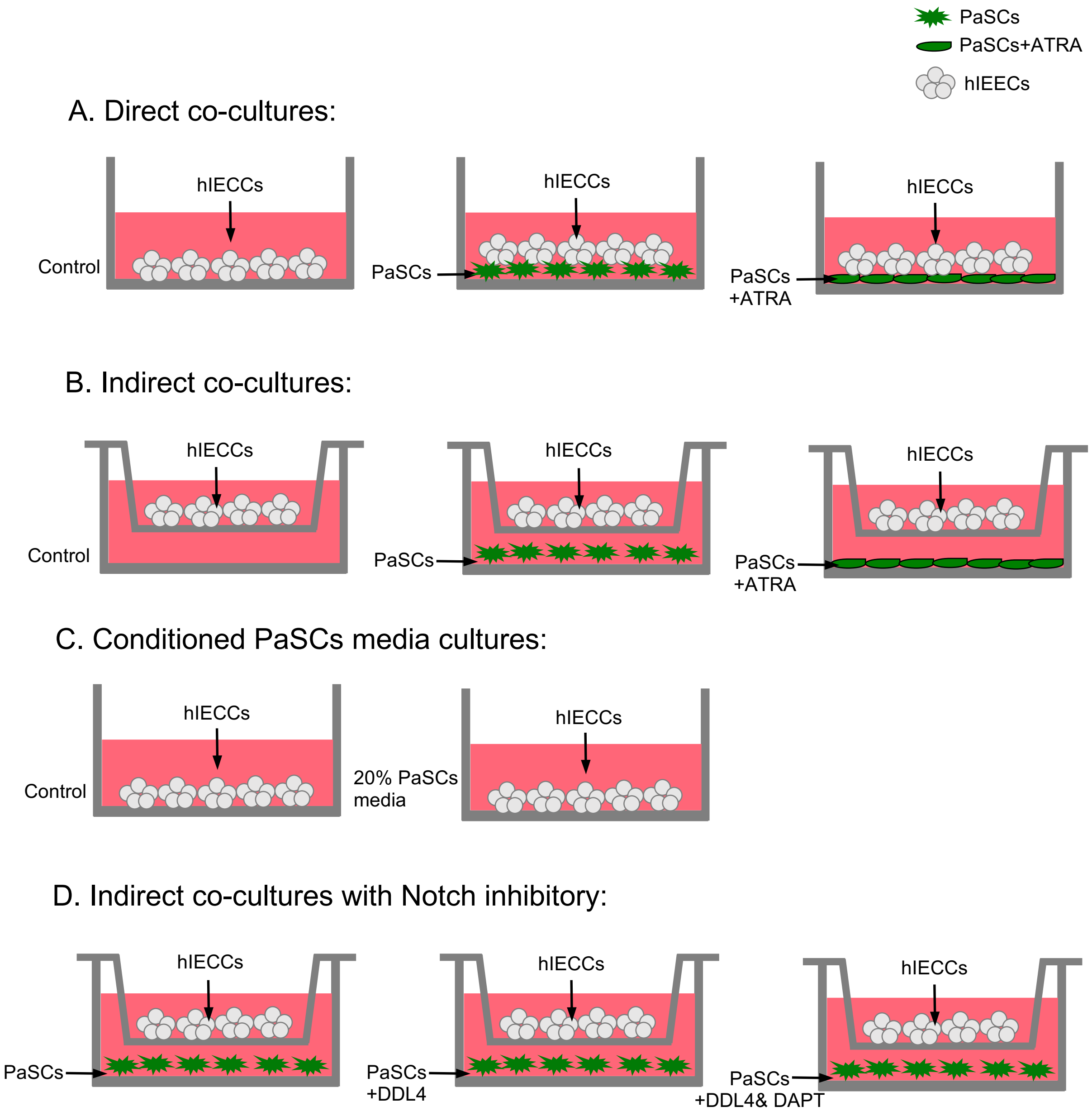

Supplementary Figure S1 Co-culture models. Direct co-cultures (**A**) and Indirect co-cultures (**B**) models. Sub-confluent (80-90%) pancreatic stellate cells (PaSCs) in a 12-well plate were pretreated with or without 100 nM ATRA, then freshly isolated human fetal islet-epithelial cell clusters (hIECCs) were plated on top or in Millicell® Inserts and co-cultured in CMRL1066 media containing 5% FBS for 48 hours*.* (**C**) 20% Conditioned PaSCs media culture. (**D**) Indirect co-cultures with Notch inhibition.

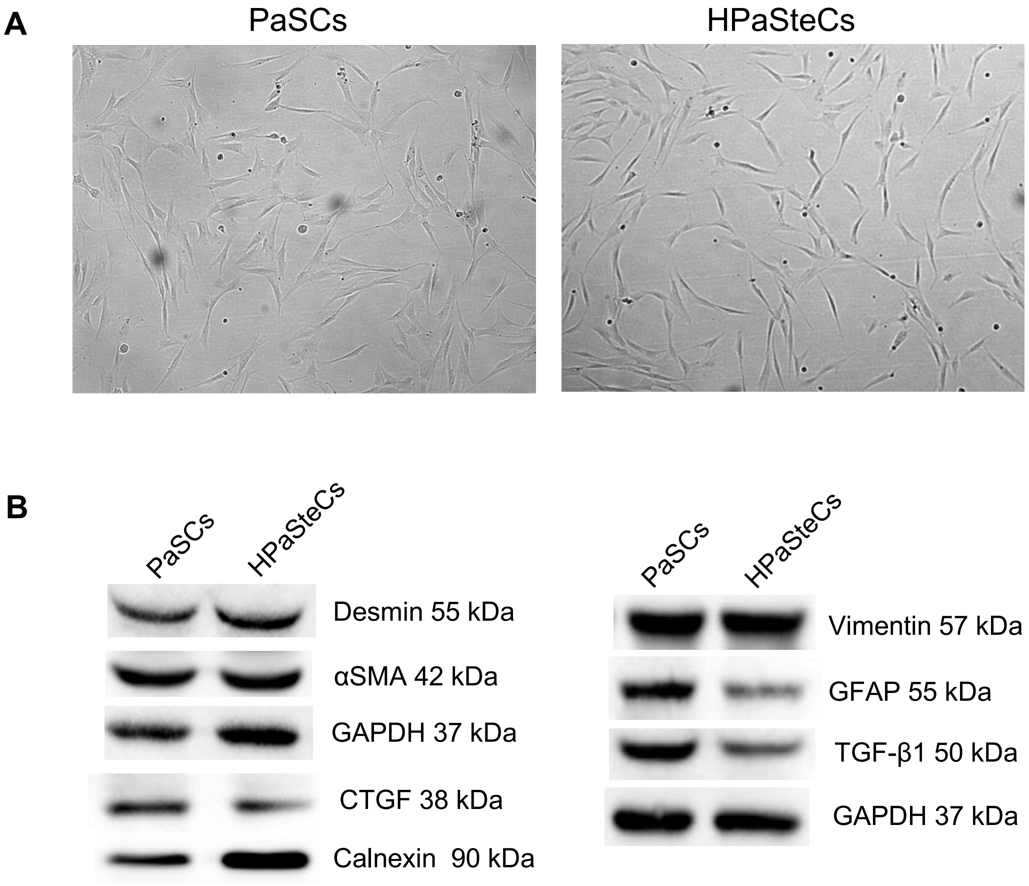

Supplementary Figure S2. (A) Phase-contrast images of PaSCs isolated from human fetal pancreas in our lab and purchased HPaSteC (catalog #3830) from ScienCell Research Labs, United States. (**B**) Western blot gels present PaSC markers and growth factors of desmin, αSMA, vimentin, GFAP, TGFβ1 and CTGF from both cultured PaSCs and HPaSteC cells.

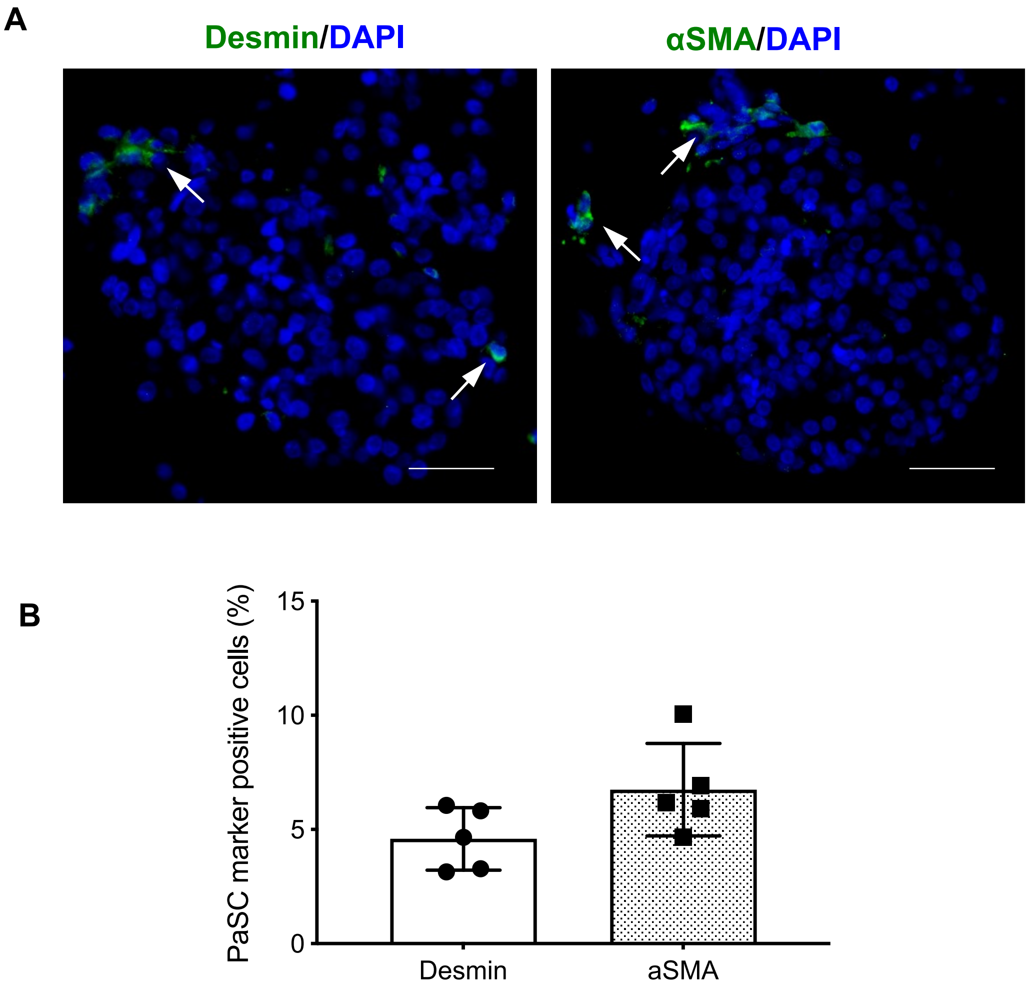

Supplementary Figure S3 (A) Freshly isolated human (18 wks of fetal age) fetal islet-epithelial cell cluster (hIECC) containing a few PaSCs stained with desmin or αSMA (green, arrows); nuclei labelled by DAPI (blue). Scale bar: 25μm. (**B**) The percentage of desmin+ and αSMA+ cells present in the hIECC was quantified.

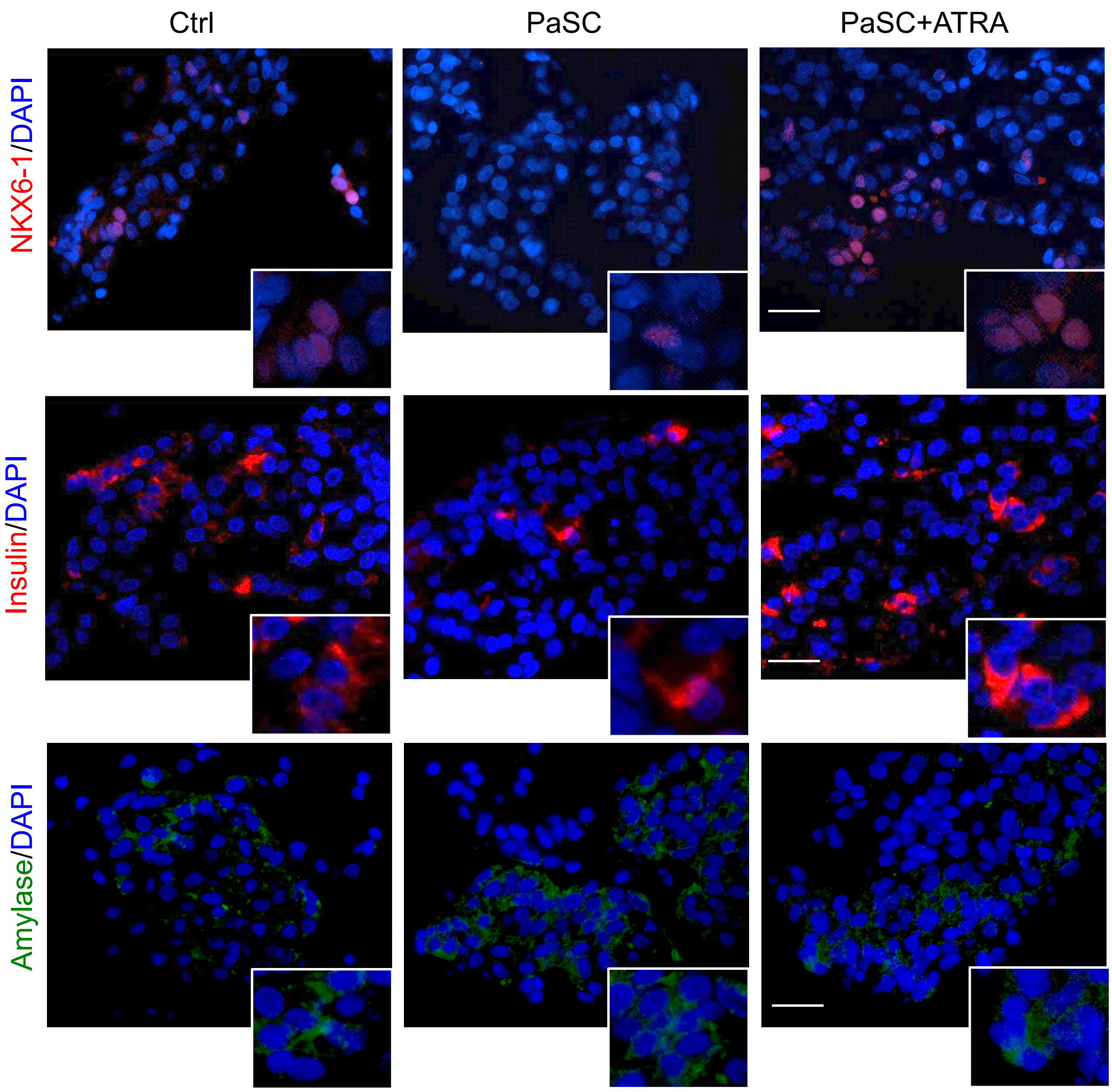

Supplementary Figure S4. Representative immunofluorescence staining images for NKX6-1, insulin (red) or amylase (green) in hIECCs during a direct co-culture with PaSCs for 48 hours, and the nuclear stain DAPI (blue). Scale bar: 25µm. Magnified images are shown in insets.

A

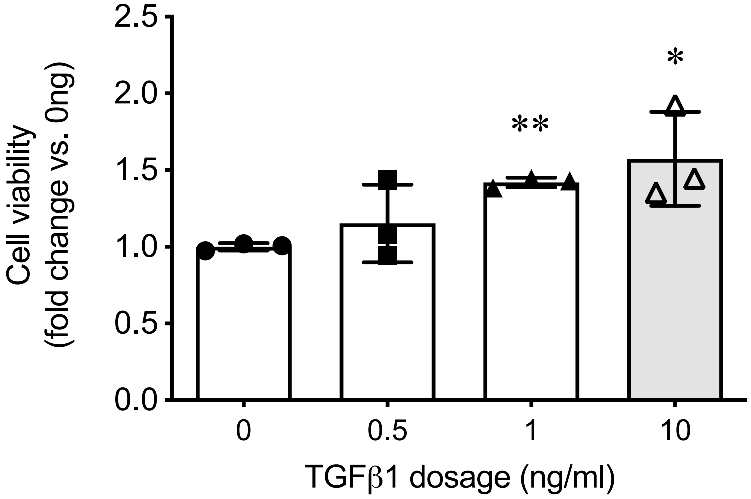

B

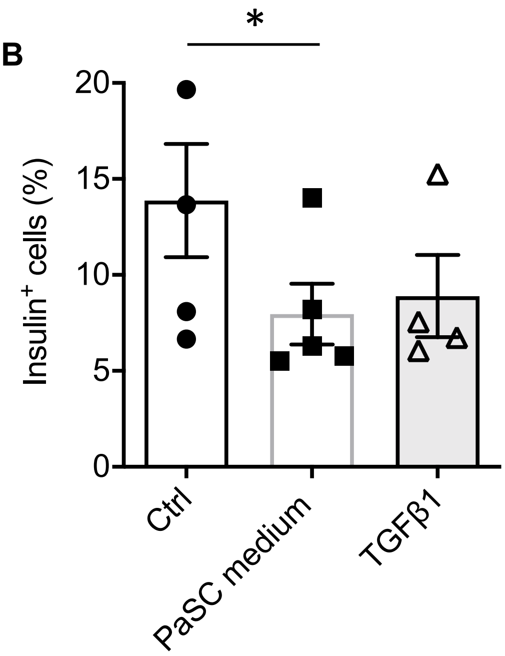

Supplementary Figure S5 (A) PaSCs cell viability by MTT showed a significantly increase with 1-10ng/ml TGFβ1 treatment. *$p<0.05$, **$p<0.01$ vs. 0ng/ml. (**B**) The percentage of insulin^{+} cells in hIEECs cultured with 20% PaSCs conditional medium (closed squares) and 10ng/ml TGFβ1 (opened triangle) compared to control (closed circle). Data are expressed as mean ± SEM (*n=3-5* experiments/treatment group).
