# Supplementary material for: Activation of Pancreatic Stellate Cells Is Beneficial for Exocrine but Not Endocrine Cell Differentiation in the Developing Human Pancreas
Source: Front Cell Dev Biol. 2021 Aug 18;9:694276. doi: 10.3389/fcell.2021.694276 (PMC8418189; doi:10.3389/fcell.2021.694276)
Supplement: Supplementary Table 1 — Antibodies for immunohistochemistry, immunofluorescence and western blot analyses. [file Table_1.DOCX]

**SUPPLEMENTARY TABLES**

**Table S1: Antibodies for immunohistochemistry/immunofluorescence and western blot analyses**

| **Primary Antibody** | | | **Source & Isotype** | | | **Dilution** | **Company** | |
| --- | --- | --- | --- | --- | --- | --- | --- | --- |
| Amylase | | rabbit polyclonal IgG | | | | 1:500 | Chemicon, Temecula, CA, USA | |
| Smooth Muscle Actin (α-SMA) | | mouse monoclonal IgG | | | | 1:100  1:1000^o^ | Abcam Inc, Cambridge, MA, USA | |
| Cytokeratin19 (CK19) | | rabbit polyclonal IgG | | | | 1:50 | Dako, Mississauga, ON, Canada | |
| Calnexin | | mouse monoclonal IgG1 | | | | 1:1500^o^ | BD Biosciences Mississauga, Canada | |
| Collagen I | | rabbit polyclonal IgG | | | | 1:50 | Santa Cruz Biotechnology, CA, USA | |
| CTGF(CCN2) | | rabbit polyclonal IgG | | | | 1:1000^o^ | Abcam Inc, Cambridge, MA, USA | |
| Desmin | rabbit polyclonal IgG | | | | | 1:200 | Abcam Inc, Cambridge, MA, USA | |
| Cyclin D1 | | mouse monoclonal IgG2a | | | | 1:2000^o^ | Cell Signaling Technology Inc, Danvers, MA, USA | |
| Fibronectin | | mouse monoclonal IgG1 | | | | 1:100 | Chemicon, Temecula, CA, USA | |
| GAPDH | | rabbit polyclonal IgG | | | | 1:2000^o^ | Santa Cruz Biotechnology, CA, USA | |
| Glucagon | | mouse monoclonal IgG1 | | | | 1:1500 | Sigma, Saint Louis, Missouri, USA | |
| Insulin | | mouse monoclonal IgG1 | | | | 1:1500 | Sigma, Saint Louis, Missouri, USA | |
| Ki67 | | mouse monoclonal IgG1 | | | | 1:300^a^ | BD Biosciences Mississauga, Canada | |
| Laminin | | rabbit polyclonal | | | | 1:50 | Chemicon, Temecula, CA, USA | |
| NKX6.1 | | mouse monoclonal IgG1 | | | | 1:100^a^ | DHSB, Iowa City, IA, USA | |
| PECAM (CD31) | | rabbit polyclonal IgG | | | | 1:50 | Santa Cruz Biotechnology, CA, USA | |
| SOX9 | | mouse monoclonal IgG2b | | | | 1:100^a^ | Abcam Inc, Cambridge, MA, USA | |
| TGFβ1 | | rabbit polyclonal | | | | 1:1000^o^ | Sigma, Saint Louis, Missouri, USA | |
| Vimentin | | mouse monoclonal IgG1 | | | | 1:1000^o^ | Santa Cruz Biotechnology, CA, USA | |
|  | | | |  |  | | |  |

^o^ Dilution selected for optimal western blot probing

^a^ Citrate (pH 6.0) antigen retrieval used

**Table S2: Sequences of primers used in Real-time RT-PCR assays**

| **Primer Name** | **Accession Number** | **Primer Pair Sequence 5’----3’**  **(*Sense/Antisense*)** | **Location**  **(*nt*)** | | **Fragment**  **Size (bp)** |
| --- | --- | --- | --- | --- | --- |
| **AMY1A** | NM_004038.3 | TGC TGG GCT CAG TAT TCC TCA  CCC TTG GGA GCT AAA TAT CGC | 332-352  447-427 | | 116 |
| **DES**  (DESMIN) | NM_001927.3 | CCA GTC CTA CAC CTG CGA GAT  GCA ATG TTG TCC TGG TAG CC | 1070-1090  1189-1170 | | 120 |
| **GCG**  (GLUCAGON) | NM_002054.4 | GAT GAA CGA GGA CAA GCG CC  CCT TTC ACC AGC CAA GCA AT | 394-413  633-614 | | 240 |
| **HES1** | NM_005524.3 | GTC AAC ACG ACA CCG GAT AAA  AGT GCG CAC CTC GGT ATT AAC | 300-320  650-630 | | 351 |
| **INS**  (INSULIN) | NM_000207.1 | TCA CAC CTG GTG GAA GCT CTC TA  ACA ATG CCA CGC TTC TGC AGG GAC | 156-178  334-311 | | 179 |
| **NEUROG3**  (NGN-3) | NM_020999.3 | AGC CGG CCT AAG AGC GAG TT  TTG GTG AGC TTC GCG TCG TC | 451-470  608-589 | | 158 |
| **NKX6-1** | NM_006168.2 | AGA CCC ACT TTT TCC GGA CA  CCG CTG CTG GAC TTG TGC TT | 363-382  1058-1039 | | 335 |
| **NOTCH1** | NM_017617.3 | AAC TGC CTG CTG CCC TAC AC  CTC ATA GTC CTC GGA TTG CC | 2446-2465  2553-2524 | | 108 |
| **NOTCH2** | NM_024408.3 | GCG CCA GCC TCC TTA TTA CT  ACG CCA TCC CGA GCT TTG TC | 4473-4492  4610-4591 | | 138 |
| **JAG1** | NM_000214.2 | GAA CGG TGC CCA GTG CTA CAA  AAT TTG CCT CCC GAC TGA CT | 2121-2141  2354-2335 | | 234 |
| **PDX1**  (IPF1) | NM_000209.3 | CTC CTA CAG CAC TCC ACC TTG  CCG AGT AAG AAT GGC TTT ATG | 1262-1282  1414-1394 | 153 | |
| **PTF1A** | NM_178161.3 | AGA CGC CTT TCC TTC TTC GT  CGG TAG CAG TAC TCG TGG AG | 258-277  407-388 | | 140 |
| **18S** | NR_003286.1 | GTA ACC CGT TGA ACC CCA TTC  CCA TCC AAT CGG TAG TAG CG | 1577-1597  1729-1710 | | 153 |
|  |  |  |  | |  |

**Table S3. List genes for pancreatic stellate cell markers and associated notch signaling expressed during human fetal pancreatic development**

| **Genbank**  **(Probe ID)** | **Gene symbol** | **Description** | **14-16 weeks** | **19-21 weeks** |
| --- | --- | --- | --- | --- |
| NM_002055  (203540_at) | GFAP | Glial fibrillary acidic protein | **1.08 +/- 0.05** | **1.18 +/- 0.06** |
| NM_003380  (201426_s_at) | VIM | Vimentin | 0.58 ± 0.09* | 0.45 ± 0.02** |
| NM_024609  (218678_at) | NES | Nestin | 0.63 ± 0.13* | 0.50 ± 0.05** |
| NM_001927  (202222_s_at) | DES | Desmin | 0.67 ± 0.07* | 0.70 ± 0.06* |
| NM_001613  (200974_at) | ACTA2^a^ | Actin, alpha 2, smooth muscle | 0.66 ± 0.11* | 0.45 ± 0.03*** |
| NM_017617 (218902_at) | NOTCH1 | Notch homolog 1 | 0.77 ± 0.05* | 0.63 ± 0.03*** |
| AU158495  (212377_s_at) | NOTCH2 | Notch homolog 2 | 0.63 ± 0.09* | 0.49 ± 0.02*** |
| U61276  (209098_s_at) | JAG1 | jagged 1 | 0.65 ± 0.06* | 0.59 ± 0.03** |
| NM_005524 (203395_s_at) | HES1 | Hairy and enhancer of split 1 | 0.83 ± 0.003 | 0.73 ± 0.04* |

Biological replicates: 8–10 weeks (n=4), 14–16 weeks (n=5) and 19–21 weeks (n=6)

Data are expressed as mean fold changes ± SEM and normalised using the RMA to show expression relative to 8–10 weeks data. *p<0.05; **p<0.01; ***p<0.001 vs. 8-10 weeks group ( Lyttle et al., 2008). ^a^Also known as ⍺SMA
